# Keloidal Scleroderma: Case Report and Review

**DOI:** 10.1155/2015/635481

**Published:** 2015-11-30

**Authors:** Sama Kassira, Tarannum Jaleel, Peter Pavlidakey, Naveed Sami

**Affiliations:** Department of Dermatology, University of Alabama at Birmingham, EFH 414, 1530 3rd Avenue S, Birmingham, AL 35294, USA

## Abstract

*Objective*. We report a rare case of keloidal scleroderma and provide an analysis of similar cases.* Results*. A 41 year-old woman presented with dark brown, indurated, exophytic nodules over the chest along with smaller hyperpigmented plaques scattered over the abdomen, with concomitant sclerodactyly. The clinical, laboratory, and pathological findings were consistent with a diagnosis of keloidal scleroderma. The patient was treated with methotrexate, resulting in reduced firmness of her plaques and no new lesions. A literature review of previously reported cases was performed using keywords including keloidal morphea, keloidal scleroderma, nodular morphea, and nodular scleroderma. In our review, the majority of patients were African American and female. 91% of cases had nodular lesions with distribution on the trunk. The majority of patients exhibited sclerodactyly and pulmonary involvement was reported in 28%1. The majority of patients were ANA positive (63%) and only 10% demonstrated anti-SCL-70 positivity.* Conclusion*. Keloidal scleroderma is a rare presentation, which can often be clinically confused with keloid and scar formation. Due to this being a rare variant, our knowledge of treatment options and efficacy is limited. Methotrexate could be considered as an initial treatment option for patients with progressive keloidal scleroderma.

## 1. Introduction

Keloidal scleroderma is a very rare diagnosis, which has been also reported with alternate nomenclature including keloidal morphea, nodular morphea, and nodular scleroderma. Keloidal scleroderma presents as multiple keloid-like lesions that occur in the absence of preceding trauma or injury and can be associated with localized or systemic symptoms of scleroderma. This is in contrast to the flat and/or depressed plaques with associated tightening of the skin that is seen in classical cutaneous scleroderma. Histopathological findings in keloidal scleroderma can be variable. We report a 41-year-old woman with keloidal scleroderma and provide a review of 43 reported cases of this variant of scleroderma.

## 2. Case

A 41-year-old African American woman presented with initial symptoms of burning and stinging of the upper body for over 4 weeks, progressing to dark firm painful areas on her chest, neck, and abdomen with concomitant sclerodactyly. She denied tightening around her mouth, dry eyes or mouth, arthritis, dysphagia, or signs of Raynaud's phenomenon.

**Figure 1:**
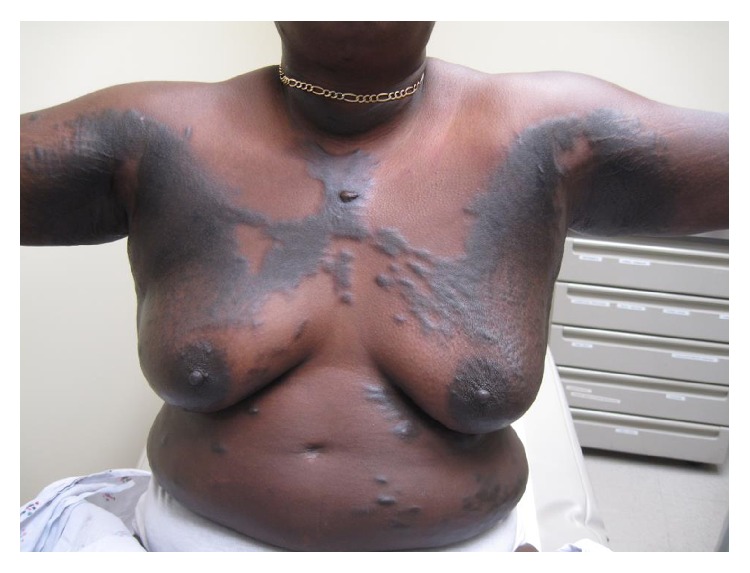
Keloidal scleroderma. Multiple, scattered, and hyperpigmented nodules overlying plaques along the trunk and upper extremities.

Physical examination revealed dark brown indurated nodules with a slightly violaceous border over the chest and breasts along with smaller hyperpigmented plaques scattered over the abdomen (Figure 1). There was also extensive hyperpigmentation and skin tightening over the anterior neck, chest, axillae, and abdomen. A hypertrophic, exophytic papule overlying a hyperpigmented plaque was present over the center of the chest. Examination of the hands showed a contracture of the left hand 4th and 5th digits with slight tapering of the fingertips. There was sparing of the face and telangiectasias were absent.

Complete blood count, metabolic panel, and hepatitis serologies did not reveal any abnormalities. Serum ANA (antinuclear antibody) titer was elevated (1 : 1280). Anti-SSA and anti-SSB serum antibodies were both elevated, while SCL70 and anti-Smith autoantibody titers were within normal limits.

**Figure 2:**
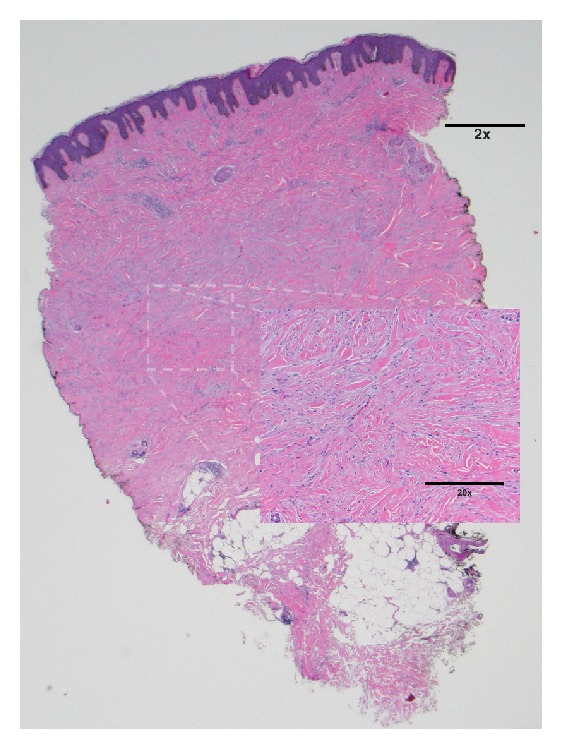
Keloidal scleroderma. Histologic sections show an acanthotic epidermis with overlying basilar hyperpigmentation. Within the dermis there is a proliferation of myofibroblasts and thickened collagen bundles. There is a lack of vertically oriented blood vessels and a lack of atrophy of the overlying epidermis speaking against that of a keloid or scar. At low power (2x) biopsy has a barrel-shaped appearance. The dermal component is expansile and extends beyond that of the epidermal component.

**Figure 3:**
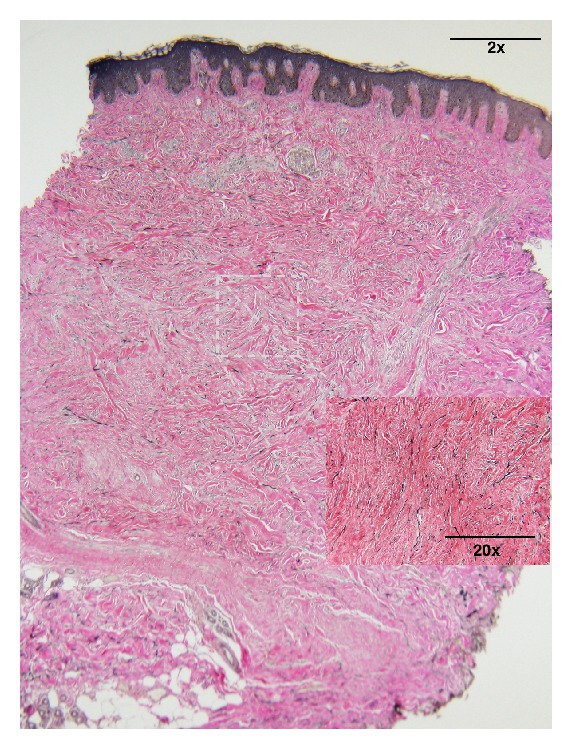
A tissue elastic stain shows preserved elastic fibers within areas of scleroderma. In areas of keloid these elastic fibers are typically absent, thus supporting the diagnosis of keloidal scleroderma and not that of a keloid.

Histologic sections show an acanthotic epidermis with overlying basilar hyperpigmentation. Within the dermis there is a proliferation of myofibroblasts and thickened collagen bundles. There is a lack of vertically oriented blood vessels and a lack of atrophy of the overlying epidermis speaking against that of a keloid or scar. At low power biopsy has a barrel-shaped appearance. The dermal component is expansile and extends beyond that of the epidermal component (Figure 2). A tissue elastic stain shows preserved elastic fibers within areas of scleroderma (Figure 3). In areas of keloid these elastic fibers are typically absent, thus supporting the diagnosis of keloidal scleroderma and not that of a keloid.

The clinical and pathological findings were consistent with a diagnosis of keloidal scleroderma. The patient was treated with methotrexate (17.5 mg/week) for six weeks resulting in reduced firmness of her plaques and no new lesions.

## 3. Discussion

Keloidal scleroderma is a rare presentation, which can often be clinically confused with keloid and scar formation. Although there have been some reports suggesting that keloidal scleroderma may represent two distinct processes with keloid formation causally unrelated to sclerosis, others suggest that there is a combined mechanism with a dermal inflammatory process of sclerosis forming keloidal lesions [1]. High levels of tenascin have been histologically observed in keloidal scleroderma lesions as well as increased levels of TFG-beta cytokines [1, 2]. Tenascin has been shown to have distinct mid-dermal distribution in sclerodermal lesions, reflecting active fibrosis [1]. A strikingly different tenascin distribution in the nodular lesions of a single case as compared to sclerotic tissue has been shown, suggesting a differing pathological course between the nodular and sclerotic lesions [1]. However, keloidal scleroderma lesions have shown increased levels of TGF-beta and connective tissue growth factor (CTF), which is similarly seen in fibroblasts of classic sclerodermal lesions suggesting analogous pathogenesis of collagen synthesis [1, 2].

We performed a literature review for previously reported cases using keywords, which have been used to describe similar clinical presentations, including keloidal morphea, keloidal scleroderma, nodular morphea, and nodular scleroderma. All cases where the clinical presentation was confirmed by a histopathological diagnosis were included. Clinical data of 43 patients from 29 different publications is presented in Table 1.

In our review, the majority of patients were African American and female. Ages ranged from 3 to 70 years (median 41). 91% of cases had nodular lesions with distribution on the trunk, while one case had lesions in the intertriginous areas. The majority of patients presented with sclerodactyly as well as extracutaneous manifestations of systemic scleroderma (Table 1). Pulmonary involvement was reported in 28% and renal involvement in 5% [1]. Ten percent of cases noted an external trigger prior to the onset of keloidal plaques, including infection, D-penicillamine, tetanus vaccine, and environmental exposures. One patient, who already had a diagnosis of keloidal scleroderma, did not have any involvement at a recent surgical site [1].

Laboratory values demonstrated the majority of patients were ANA positive (63%) and only 10% demonstrated anti-SCL-70 positivity. A single case noted positive anti-*Borrelia* antibodies and two cases were seropositive for anti-SSB [1].

Current treatments for cutaneous and systemic sclerosis include topical or intralesional corticosteroids, a topical vitamin D analog, topical tacrolimus or imiquimod, UV light therapy, methotrexate, and systemic steroids [3–5].

Thirteen out of 22 cases were treated with local and/or systemic steroids. The majority reported no response to treatment. Only three cases showed partial response with local and/or systemic steroid therapy and one case showed complete response to systemic steroids. D-penicillamine was used in 6 patients and only one patient had full resolution with 5 years of oral D-penicillamine in combination with topical steroids. However, one patient showed progression while on therapy [1, 6–9]. Both patients who showed complete response to D-penicillamine and systemic steroids had systemic scleroderma with pulmonary involvement [1]. Azathioprine (100 mg daily) was also used in a case for an unknown duration with no resolution. Surgical removal of several large nodules showed complete resolution in one patient [1, 10]. Multiple studies using PUVA showed partial response. However, one case using PUVA in combination with methotrexate and systemic steroids reported no response [2]. Our patient reported a partial response with six weeks of methotrexate with a decrease in firmness of the keloidal plaques and no new active lesions.

In conclusion, diagnosis of scleroderma should be considered in a patient with extensive keloids. Due to this being a rare variant, our knowledge of treatment options and efficacy is limited. Methotrexate could be considered as an initial treatment option for patients with progressive keloidal scleroderma. Further research is still needed to understand the pathogenesis and treatment of this rare variant of scleroderma.

## Figures and Tables

**Table 1:** Patient characteristics, clinical manifestations, and laboratory findings in keloidal scleroderma.

Patient characteristics	% of total patients
Gender	
Female	70
Male	30
Race	
African American	59
Caucasian	30
Hispanic	7
Middle Eastern	4
Clinical manifestation	58
Sclerodactyly	58
Raynaud's	47
Arthritis	30
Pulmonary involvement	28
Esophageal dysmotility	23
Renal involvement	5
Distribution	
Trunk	91
Acral	60
Head/neck	35
Intertriginous	2
External trigger	10
Laboratory results	
ANA	63
Elevated ESR	15
Anti-Scl-70	10

## Abbreviations

ANA: Antinuclear antibody
SSA: Anti-Ro antibody
SSB: Anti-La antibody
TGF: Transforming growth factor
CTF: Connective tissue factor
UV: Ultraviolet
PUVA: Psoralen plus Ultraviolet-A treatment.
